# A pancancer analysis of the clinical and genomic characteristics of multiple primary cancers

**DOI:** 10.1038/s41598-024-52659-3

**Published:** 2024-01-29

**Authors:** Baiwen Zhang, Lina He, Cong Zhou, Xiaojiao Cheng, Qingli Li, Yao Tang, Fuli Li, Tinglei Huang, Shuiping Tu

**Affiliations:** Department of Oncology, State Key Laboratory of Systems Medicine for Cancer, Renji Hospital, Shanghai Jiao Tong University School of Medicine, Shanghai, China

**Keywords:** Cancer, Oncology

## Abstract

Multiple primary cancer (MPC) denotes individuals with two or more malignant tumors occurring simultaneously or successively. Herein, a total of 11,000 pancancer patients in TCGA database (1993–2013) were divided into MPC or non-MPC groups based on their history of other malignant tumors. The incidence of MPC has risen to 8.5–13.1% since 2000. Elderly individuals, males, early-stage cancer patients, and African Americans and Caucasians are identified as independent risk factors (p < 0.0001). Non-MPC patients exhibit significantly longer overall survival (OS) and disease-free survival (DFS) (p = 0.0038 and p = 0.0014). Age (p < 0.001) and tumor staging at initial diagnosis (p < 0.001) contribute to this difference. In our center, MPC was identified in 380 out of 801 tumor events based on SEER criteria. The peak occurrence of secondary primary was about 1–5 years after the first primary tumor, with a second small peak around 10–15 years. Multiple tumors commonly occur in the same organ (e.g., breast and lung), constituting 12.6%. Certain cancer types, notably skin cutaneous melanoma (SKCM), exhibit significantly higher tumor mutational burden (TMB) in the MPC group (17.31 vs. 6.55 mutations/MB, p < 0.001), with high TMB associated with improved survival (p < 0.001). High TMB in MPC may serve as a predictor for potential immunotherapy application.

## Introduction

Multiple primary cancer (MPC) refers to the presence of two or more malignant tumors in the same or different organs or tissues simultaneously or successively. These tumors, pathologically and histologically considered cancers from different primary sites, are mainly distinguishing them from tumor recurrence and metastasis^1^. MPC can be classified into synchronous multiple primary cancer (SMPC) or metachronous multiple primary cancer (MMPC) based on the time interval between the second tumor and the primary tumor. The diagnosis of MPC has evolved, and two main criteria, established by the Surveillance Epidemiology and End Results (SEER) Program and the International Association of Cancer Registries and International Agency for Research on Cancer (IACR/IARC) organization, are commonly used in studies^2^. Differences in these criteria include the understanding of the primary site and the definition of SMPC and MMPC^2^. In this study, we utilized SEER as the main diagnostic criteria.

At present, the incidence of multiple primary tumors in different studies ranges from 1.63 to 10.9%^1,3–6^. The risk of developing multiple primary tumors increases with a longer follow-up duration, reaching 4.3%, 7.7%, and 12.4% over an average follow-up period of 5, 10, and 20 years, respectively^7^. This heightened risk is primarily associated with age, as evidenced by a significant increase in incidence from 1% for a 30-year-old person to 18% for a 70-year-old person^8^. Individual with a history of cancer face a 14% increased risk of getting another primary tumor compared to the general population^9^. The risk of tumor recurrence varies depending on the type of first primary tumor, with a 1% recurrence risk for primary liver cancer and 16% for primary bladder cancer^10^. Factors influencing the incidence of multiple primary tumors include the location of the first primary tumor, age at initial diagnosis, environmental exposure factors, genetic factors, and previous treatment^1^.

Herein, we analyzed clinical and genomic data from large-scale sequencing data in The Cancer Genome Atlas (TCGA) database to enhance understanding and identify effective strategies for preventing and managing multiple primary tumors, ultimately improving prognosis and therapeutic methods.

## Materials and methods

### Data acquisition

We accessed the latest TCGA data stored in the Genomic Data C commons (GDC) through its website (https://portal.gdc.cancer.gov/repository) by selecting Maf format files, resulting in 132 mutation files from 33 different types of cancer. We specifically selected MuTect series files and downloaded tools provided by the GDC to aid in downloading the appropriate files.

### Data processing and analysis

Data processing and analysis in this study were performed using the following packages in R (Version 4.2.0): ggplot, readr, dplyr, survminer, and survival.

In the consolidated datasets of 33 cancer types in TCGA database, screening was performed according to HISTORY_OTHER_MALIGNANCY, resulting in 10,016 effective cases. HISTORY_OTHER_MALIGNANCY was defined as “Yes”, “Yes, History of Prior Malignancy”, or “Yes, History of Synchronous/Bilateral Malignancy” for multiple primary malignancy group (n = 974), as “No” for non-multiple primary malignancy group (n = 9042). The age, sex, race, and pathological stage were statistically described to compare their differences. The pathological staging was based on the AJCC_PATHOLOGIC_TUMOR_STAGE divided into stages I-IV, and the individuals classified as stage 0 and unable staging X were removed.

Survival analysis was performed based on OS_STATUS and DFS_STATUS. There were 9619 effective cases for overall survival, comprising 919 MPC cases and 8700 non-MPC cases. Data from 8290 patients with disease-free survival (DFS) were obtained, including 783 MPC patients and 7507 non-MPC patients.

### Cases enrolled in our center

Multiple cases of primary tumors diagnosed at the Renji Hospital, Shanghai Jiao Tong University School of Medicine from January 2017 to December 2022 were included. The diagnostic criteria for MPC followed the SEER project. A total of 380 patients were recruited and followed up. Collected information included sex, age, location, pathological diagnosis, and time of diagnosis of the first, second, and later primary tumors. This was an observational study approved by the Ethics Committee of Renji Hospital Affiliated to Shanghai Jiao Tong University School of Medicine, and written informed consent was obtained from every patient. All authors confirmed that all methods of this study were performed in accordance with the relevant guidelines and regulations.

### Statistical methods

Statistical analysis was conducted by R (Version 4.2.0). T tests were used to compare the means of continuous variables with normally distributed continuous variables between two groups, while the Wilcoxon rank sum test was utilized for continuous variables with non-normal distribution. The chi-square test or Fisher’s exact test was applied to compare different classification variables. The survival analysis of the groups was performed by Kaplan‒Meier survival curves, and Cox regression and logistic regression were used for statistical tests. A significance level of p < 0.05 was considered statistically significant.

### Ethics declarations

The study was approved by the Ethics Committee of Renji Hospital Affiliated to Shanghai Jiao Tong University School of Medicine (Shanghai, China) (Approval number: RA-2022-650), and written informed consent was obtained from every patient. All authors confirmed that all methods of this study were performed in accordance with the relevant guidelines and regulations.

## Results

### Incidence of multiple primary cancer

**Figure 1:**
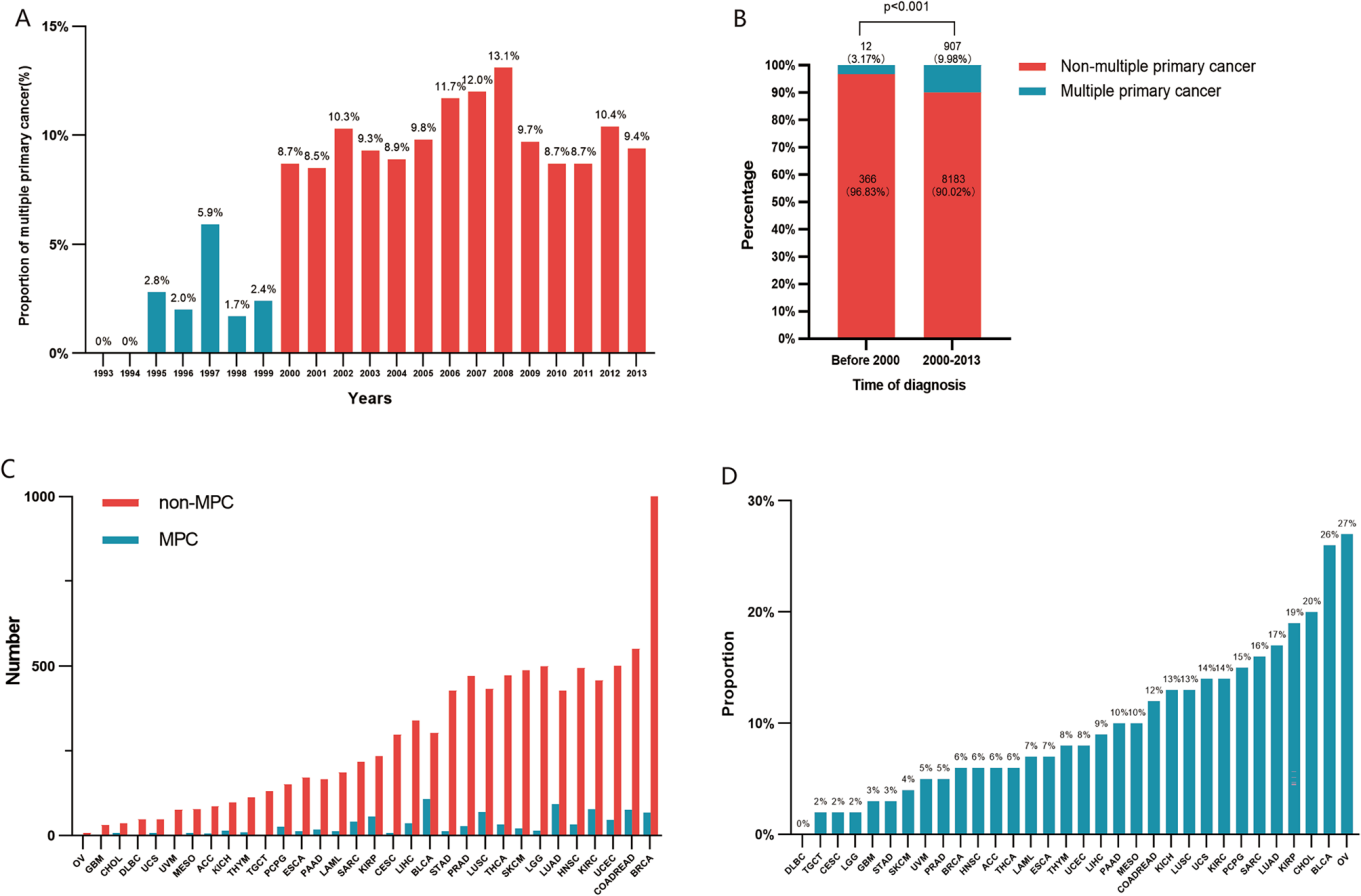
Incidence of multiple primary cancer (MPC) in TCGA database. (**A**) The proportion of MPC patients diagnosed in different years from 1993 to 2013. (**B**) The proportion of MPC and non-MPC in pan-cancer diagnosed before and after 2000. (**C**) The number of patients with MPC and non-MPC in different cancer types. (**D**) The proportion of patients with MPC in different cancer types.

We first assessed the proportion of patients with MPC diagnosed in TCGA database from 1993 to 2013 (Fig. 1A). The incidence of MPC was at a low level before 2000, fluctuating between 0 and 5.9%. Since 2000, however, the incidence has consistently stayed around 10%, ranging from 8.5 and 13.1%. Further analysis revealed that a significant increase in the proportion of MPC after 2000 accounting for 9.98% (907/9090) compared to an incidence of 3.17% (12/378) before 2000 (12/378) (p < 0.001) (Fig. 1B). In addition, the occurrence of MPC varied significantly among different tumor types, ranging from 0 to 27.3% (Fig. 1C,D). The specific data are shown in Table 1. Notably, bladder cancer (26.46%, 109/412) had a significantly high proportion of patients with previous malignant tumors, aligning with previous reports^9^.

**Table 1:** Number of patients of multiple primary cancer and non-multiple primary cancer.

Primary site	MPC n (%)	Non-MPC n (%)	OR (95% CI)	p-val
PAN-CANCER	974 (9.72%)	9042 (90.28%)	∕	∕
ACC (adrenocortical carcinoma)	6 (6.52%)	86 (93.48%)	0.65 (0.23–1.47)	0.378
BLCA (bladder urothelial carcinoma)	109 (26.46%)	303 (73.54%)	3.63 (2.86–4.59)	0.000
BRCA (breast invasive carcinoma)	68 (6.20%)	1028 (93.80%)	0.59 (0.45–0.76)	0.000
CESC (cervical squamous cell carcinoma and endocervical adenocarcinoma)	8 (2.61%)	299 (97.39%)	0.24 (0.10–0.49)	0.000
CHOL (cholangiocarcinoma)	9 (20%)	36 (80%)	2.33 (0.99–4.95)	0.037
COADREAD (colorectal adenocarcinoma)	77 (12.28%)	550 (87.72%)	1.33 (1.02–1.70)	0.031
DLBC (lymphoid neoplasm diffuse large b-cell lymphoma)	0 (0%)	48 (100%)	0 (0–0.74)	0.013
ESCA (esophageal carcinoma)	14 (7.57%)	171 (92.43%)	0.76 (0.40–1.31)	0.380
GBM (glioblastoma multiforme)	1 (3.12%)	31 (96.88%)	0.30 (0.01–7.80)	0.363
HNSC (head and neck squamous cell carcinoma)	33 (6.25%)	495 (93.75%)	0.61 (0.41–0.87)	0.005
KICH (kidney chromophobe)	15 (13.27%)	98 (86.73%)	1.43 (0.77–2.48)	0.200
KIRC (kidney renal clear cell carcinoma)	78 (14.53%)	459 (85.47%)	1.63 (1.25–2.10)	0.000
KIRP (kidney renal papillary cell carcinoma)	56 (19.24%)	235 (80.76%)	2.29 (1.66–3.10)	0.000
LAML (acute myeloid leukemia)	14 (7%)	186 (93%)	0.69 (0.37–1.20)	0.227
LGG (brain lower grade glioma)	15 (2.91%)	500 (97.09%)	0.27 (0.15–0.45)	0.000
LIHC (liver hepatocellular carcinoma)	37 (9.02%)	340 (90.19%)	1.01 (0.69–1.43)	0.929
LUAD (lung adenocarcinoma)	93 (17.82%)	429 (82.18%)	2.12 (1.66–2.69)	0.000
LUSC (lung squamous cell carcinoma)	70 (13.92%)	433 (86.08%)	1.54 (1.17–2.01)	0.002
MESO (mesothelioma)	9 (10.34%)	78 (89.66%)	1.07 (0.47–2.15)	0.855
OV (ovarian serous cystadenocarcinoma)	3 (27.27%)	8 (72.72%)	3.49 (0.60–14.56)	0.084
PAAD (pancreatic adenocarcinoma)	19 (10.27%)	166 (89.73%)	1.06 (0.62–1.72)	0.802
PCPG (pheochromocytoma and paraganglioma)	27 (15.08%)	152 (84.92%)	1.67 (1.06–2.54)	0.021
PRAD (prostate adenocarcinoma)	29 (5.8%)	471 (94.2%)	0.56 (0.37–0.82)	0.002
SARC (sarcoma)	42 (16.09%)	219 (83.91%)	1.82 (1.26–2.55)	0.001
SKCM (skin cutaneous melanoma)	22 (4.68%)	448 (95.32%)	0.44 (0.27–0.68)	0.000
STAD (stomach adenocarcinoma)	14 (3.16%)	429 (96.84%)	0.29 (0.16–0.50)	0.000
TGCT (testicular germ cell tumors)	3 (2.24%)	131 (97.76%)	0.21 (0.04–0.63)	0.001
THCA (thyroid carcinoma)	34 (6.76%)	473 (94.63%)	0.66 (0.45–0.94)	0.017
THYM (thymoma)	10 (8.06%)	114 (91.94%)	0.81 (0.38–1.56)	0.647
UCEC (uterine corpus endometrial carcinoma)	47 (8.58%)	501 (91.42%)	0.86 (0.62–1.18)	0.374
UCS (uterine carcinosarcoma)	8 (14.04%)	49 (85.96%)	1.52 (0.62–3.25)	0.260
UVM (uveal melanoma)	4 (5%)	76 (95%)	0.49 (0.13–1.30)	0.185

*MPC* multiple primary cancer, *Non-MPC* non-multiple primary cancer, *OR* odds ratio, *95% CI* 95% confidence interval, *p-val* p-value, *n* number.

### General clinical characteristics

**Figure 2:**
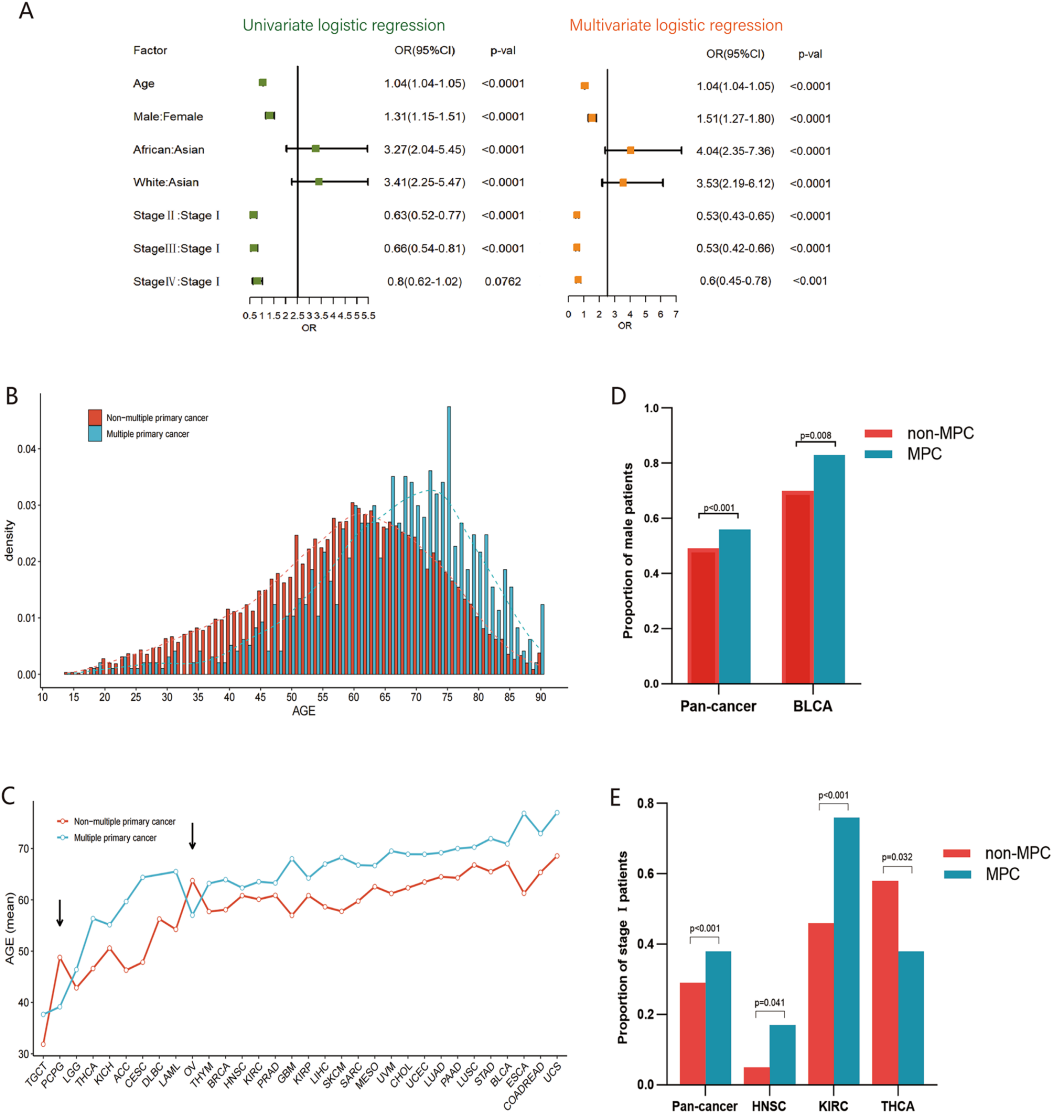
General clinical features of multiple primary tumors (MPC) in TCGA database. (**A**) Univariate and multivariate logistic regression of clinical characteristics of MPC and non-MPC. (**B**) Density distribution of age in MPC and non-MPC. (**C**) The difference in the mean age of MPC and non-MPC in different types of cancer. (**D**) The proportion of male patients in MPC and non-MPC. (**E**) The proportion of stage I patients in MPC and non-MPC in different types of cancer.

Clinical characteristics indicated significant differences in age, sex, race, and American Joint Committee on Cancer (AJCC) pathological stage between MPC and non-MPC patients in TCGA database (Table 2). Patients with MPC were older, and male patients had a higher probability of MPC than those with the primary tumor (66.00 vs. 58.39 years and 56.06% vs. 49.26%, both p < 0.001), and the proportion of MPC patients in Asia was significantly lower than that of non-MPC patients (2.47% vs. 7.91%, p < 0.001). Regarding pathological staging, the patients in stage I had a significantly higher incidence of MPC compared to non-MPC patients (38.02% vs. 29.14%, p < 0.001), while Stage II or IV patients had similar incidences of MPC, suggesting a correlation between these variables. Multivariate logistic regression analysis suggested that older age, blacks, whites, and the male population had a significantly higher risk of MPC (p < 0.001) (Fig. 2A). Patients with MPC were most likely in the early stage (p < 0.001) (Fig. 2A).

**Table 2:** Clinical characteristics of multiple primary cancer and non-multiple primary cancer in The Cancer Genome Atlas (TCGA) database.

Variables	Total (n = 10,016)	MPC (n = 974)	Non-MPC (n = 9042)	p-val
Age mean ± SD	59. 13 ± 14.57	66.00 ± 13. 12	58.39 ± 14.53	< 0.001
Gender n (%)				< 0.001
Male	5000 (49.92%)	546 (56.06%)	4454 (49.26%)	
Female	5016 (50.08)	428 (43.94)	4588 (50.74%)	
Race n (%)				< 0.001
Asian	643 (7.38%)	21 (2.47%)	622 (7.91%)	
Black	876 (10.06%)	87 (10.22%)	789 (10.04%)	
White	7193 (82.56%)	743 (87.31%)	6450 (82.05)	
Stage n (%)				< 0.001
I	2069 (30.10%)	281 (38.02%)	1788 (29.14%)	
II	2213 (32.19%)	200 (27.06%)	2013 (32.81%)	
III	1749 (25.44%)	164 (22.19%)	1585 (25.84%)	
IV	843 (12.26%)	94 (12.72%)	749 (12.21%)	

*MPC* multiple primary cancer, *Non-MPC* non-multiple primary cancer, *p-val* p-value, *n* number, *SD* standard deviation.

#### Age

In the pancancer cohort, the age of onset for MPC tended to be higher than the non-MPC cohort (Fig. 2B). While the onset age varied across different tumor types in MPC, the majority showed higher onset ages compared to that of non-MPC, with most occurring around 5–10 years later (significant differences observed in 20 of the tumor types) (Fig. 2C). Some tumor types, like cervical cancer (16.54 years) and esophageal cancer (15.59 years), exhibited age differences of more than 15 years, indicating a large distinction of age characteristics between MPC and non-MPC. Ovarian cancer (OV) and pheochromocytoma and paraganglioma (PCPG) among MPC had a lower onset age (indicated by the arrow in Fig. 2C), with statistically significant differences for PCPG (p = 0.005). In the case of young patients with PCPG, their onset may be associated with a genetic correlation of tumor syndrome^11^. Thus, while older age remains a primary factor in increased susceptibility to MPC, mutations of key genes may be the main pathogenic factor of MPC in specific tumor types.

#### Gender

In the pancancer cohort, the male‒female ratio of MPC was 1.28:1, resembling previous reports^6^. Among MPC patients, 56% (546/974) were males, significantly higher than 49% (4454/9042) in the non-MPC group (p < 0.001) (Fig. 2D). The sex differences in different tumors were not as pronounced as age differences. Excluding gender-specific tumors, the ratio of male to female in MPC was higher than non-MPC in 15 out of 26 remaining tumors, with bladder cancer showing a statistically significant difference (5.06:1 vs. 2.37:1, p = 0.008) (Fig. 2D).

#### Stage

The proportion of patients with stage I and stage II-IV tumors of MPC and non-MPC was opposite (Table 2). MPC patients exhibited a higher proportion of stage I tumors (38.02%) compared to the non-MPC population (29.14%) (p < 0.001) in the pancancer cohort (Fig. 2E). Further analysis showed a significant difference between stage I patients with MPC and non-MPC in certain cancer types, including head and neck squamous cell carcinoma (HNSC), renal clear cell carcinoma (KIRC), and thyroid cancer (THCA) (p = 0.041, p < 0.001, and p = 0.032, respectively) (Fig. 2E). Although we initially hypothesized that the higher proportion of stage I patients with MPC might result from more regular follow-up and screening, this was not observed in cancers like breast, colorectal, and lung carcinomas (p > 0.05). Notably, thyroid cancer with a relatively good prognosis and typically detectable through early screening, exhibited a higher percentage of stage II-IV in the MPC population (p < 0.05) (Fig. 2E), suggesting the need for further investigation into the specific mechanisms contributing to these findings.

### Survival analysis

**Figure 3:**
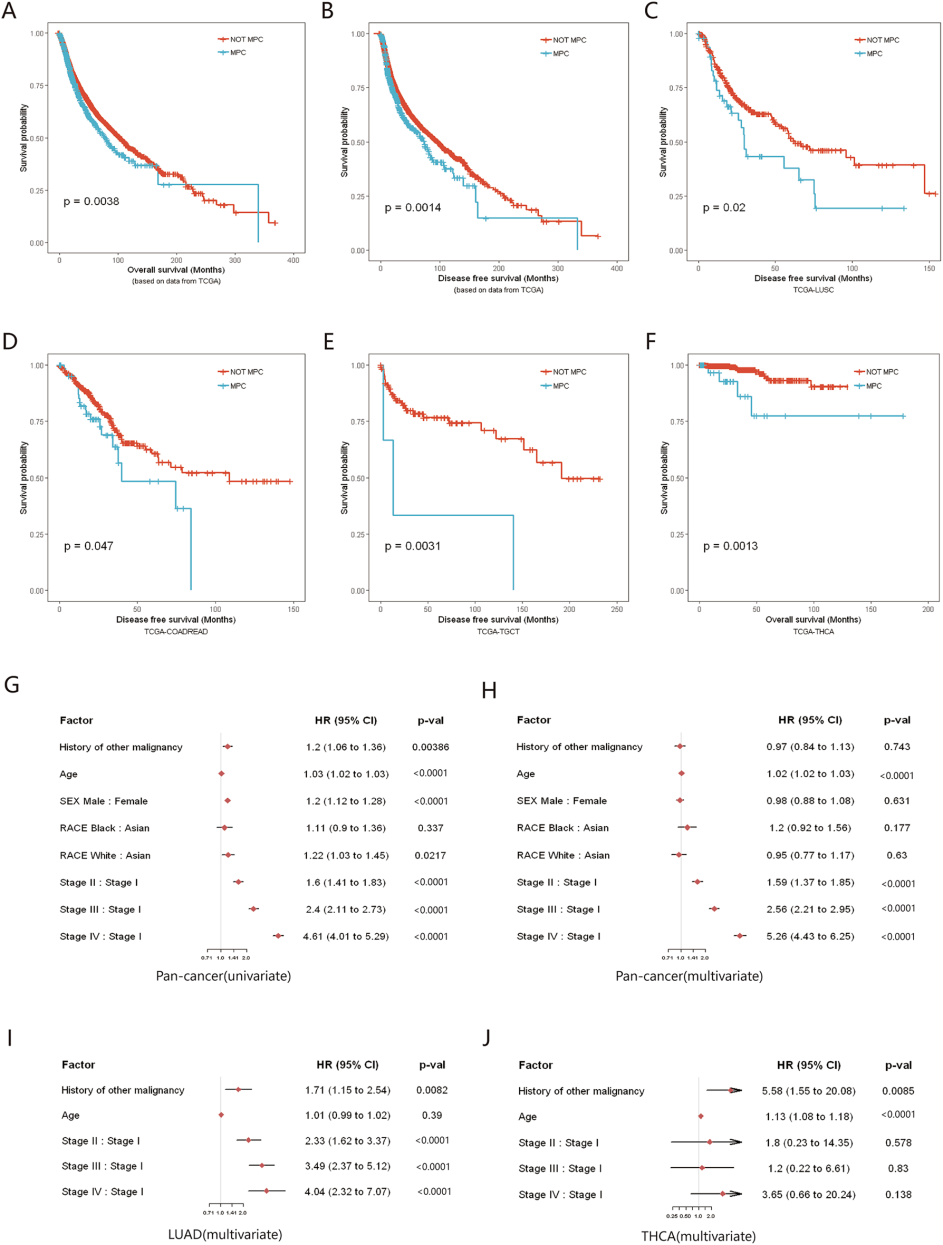
Kaplan–Meier survival curve of MPC and non-MPC, and forest plot of cox regression of survival in TCGA database. (**A–F**) The overall survival (OS) and disease-free survival (DFS) in pan-cancer datasets or different cancer types. (**G,H**) Univariate and multivariate cox regression of OS in pan-cancer datasets. (**I,J**) Multivariate cox regression of OS in lung adenocarcinoma (LUAD) and thyroid cancer (THCA) cohorts.

In this study, patients with MPC were based on a history of previous malignant tumors, using the time of onset of malignancy as a starting point to compare survival change. Non-MPC cases had significantly better overall survival (OS) and disease-free survival (DFS) than MPC cases (p = 0.0038 and p = 0.0014, respectively) (Fig. 3A,B). The median OS was 78.1 months (95% CI 67.1–99.9 months) in MPC compared to 103.5 months (95% CI 96.8–112.5 months) in non-MPC with a median DFS of 73.9 months and 97.2 months, respectively (Fig. 3A,B). Among different tumors, most MPC patients exhibited inferior survival to non-MPC patients, with some differences reaching statistical significance, such as DFS in lung squamous carcinoma (LUSC), colorectal cancer (COADREAD), and testicular germ cell tumor (TGCT) (p < 0.05) (Fig. 3C–E). Moreover, in the cohort of thyroid cancer (THCA), the OS was significantly lower in patients with MPC (p = 0.0013) (Fig. 3F). Potentially linked to the patient's first primary tumor given the long-term survival associated with thyroid cancer itself.

Univariate regression analysis indicated that a history of other malignancies affected the total survival of patients (HR 1.2, 95% CI 1.06–1.36, p = 0.0039), while it was no longer an independent risk factor for total patient survival in multivariate analysis (HR 0.97, 95% CI 0.84–1.13, p = 0.743) (Fig. 3G,H). In fact, age (HR 1.02, 95% CI 1.02–1.03, p < 0.0001) and tumor staging (stage II–IV compared with stage I, HR 1.59, 2.56 and 5.26, respectively; all p < 0.0001) were the main factors affecting survival in pancancer cohort. Exceptionally, a history of other malignancies in lung adenocarcinoma (LUAD) and thyroid cancer (THCA) significantly reduced OS in patients (p = 0.0082 and p = 0.0085, respectively) (Fig. 3I,J). In particular, for thyroid cancer, patient survival is primarily determined by the first primary cancer (HR 5.58, 95% CI 1.55–20.08).

### Synchronous and metachronous primary cancers

**Figure 4:**
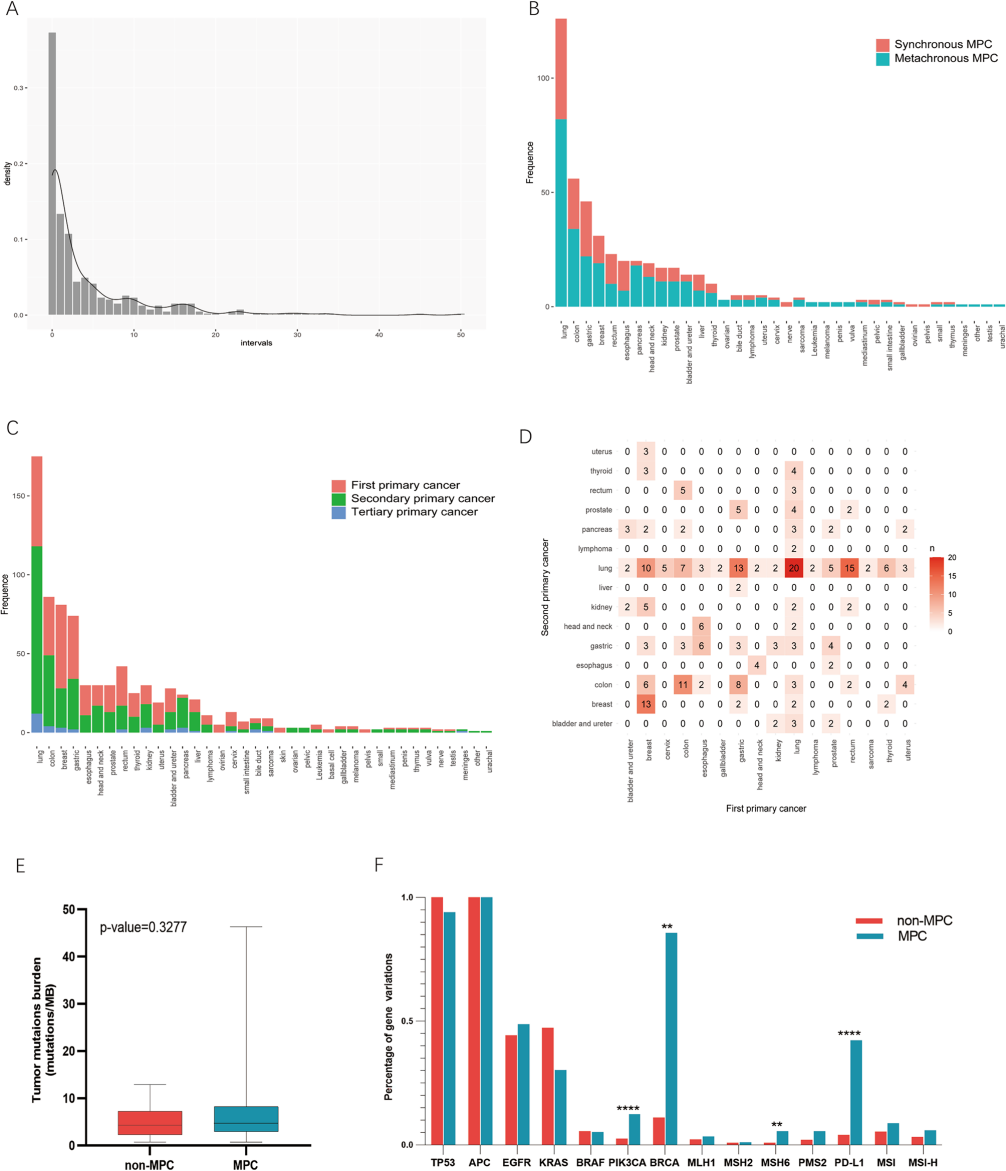
The characteristic of synchronous and metachronous multiple primary cancers in our center. (**A**) Density distribution of intervals between two cancers in metachronous MPC. (**B**) Different distribution of synchronous and metachronous MPC in the different primary sites. (**C**) Different distribution of first, secondary and tertiary primary cancers in the different primary sites. (**D**) Correlation of first and second primary cancer. (**E**) Tumor mutation burden between MPC and non-MPC patients in our cohort. (**F**) Percentage of common gene variations between MPC and non-MPC patients in our cohort.

While the TCGA data provided a comprehensive overview, limitations in information about the location and time of onset of patients’ history of other malignancies hindered the estimation of synchronous and metachronous multiple primary cancers. To address this gap, we incorporated data from 380 MPC cases in our medical center, comprising 801 tumor events with an average of 2.12 primary tumors in each patient. According to the SEER diagnostic criteria, 90 patients were identified as SMPC, and 290 patients as MMPC (Table 3). The average age of MMPC diagnosis (occurrence of the second primary tumor) was 63.00 ± 8.97 years, significantly different from SMPC (56.81 + 11.55) (p < 0.0001). The median interval between the first and second tumors in MMPC was 74.48 months, with the peak of secondary primary cancer (SPC) approximately 1–5 years from the first primary tumor and a second small peak around 10–15 years (Fig. 4A).

**Table 3:** Clinical characteristics of multiple primary cancer enrolled in our medical center.

Variable	Total (n = 380)	Synchronous (n = 90)	Metachronous (n = 290)
Age (mean ± SD)			
First primary cancer	58.49 ± 11.40	63.96 ± 8.97	56.81 ± 11.55
Second primary cancer	63.23 ± 8.96	64.01 ± 8.96	63.00 ± 8.97
Gender n (%)			
Male	207 (53.7%)	61 (67.8%)	143 (49.3%)
Female	176 (46.3%)	29 (32.2%)	147 (50.7%)
Number of primary tumors n (%)			
2 primary tumors	343 (90.3%)	85 (94.4%)	258 (89.0%)
3 primary tumors	33 (8.7%)	5 (5.5%)	28 (9.7%)
More than 3	4 (1.1%)	0 (0%)	4 (1.4%)
Interval between 2 primary tumors (months) median (IQR)	56.97 (48.54–65.56)	0.53 (0.38–0.69)	74.48 (63.88–85.81)

*n* number, *SD* standard deviation, *IQR* interquartile range.

Examining the distribution of SMPC and MMPC across various cancer sites, we observed that 65% (13/20) of esophagus cancer patients had SMPC, significantly higher than that of other tumors (p = 0.02), while 90% (18/20) of pancreas tumors were metachronous (p = 0.0089) (Fig. 4B). The difference in other cancers did not reach statistical significance (p > 0.05). Time sequence differences in tumor onset were also noted across sites (Fig. 4C). The percentage of SPC in pancreas and lung cancer exceeded that in other tumors (p < 0.0001) (Fig. 4C).

Further exploring the correlation between the first and second primary tumors, we identified common combinations such as lung-lung (20 cases), rectum-lung (15 cases), gastric-lung (13 cases), and breast-breast cancers (13 cases) (Fig. 4D). In various combinations, the second and first primary tumors in the same organ or system were the most frequent, constituting 48/380 (12.6%) of cases, similar to previous findings^5^.

### Genomic characteristics

#### MPC has significant higher tumor mutation burden than non-MPC

**Figure 5:**
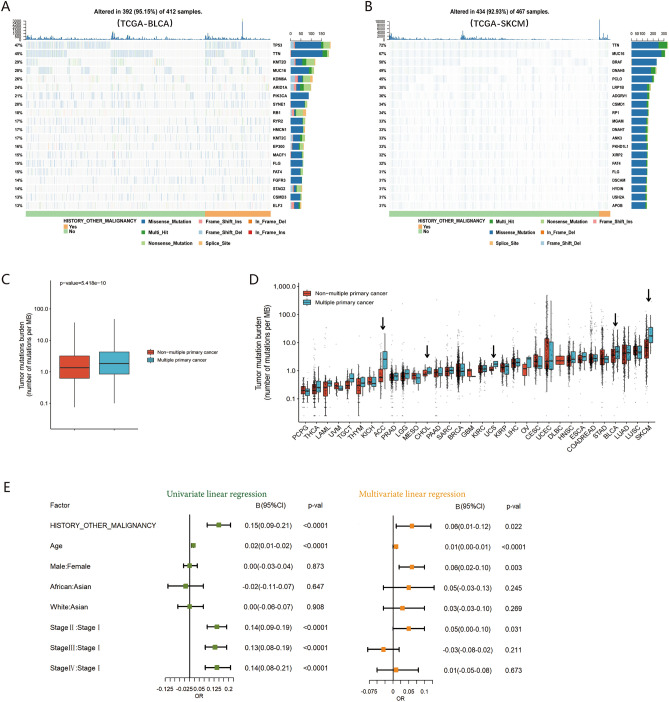
The tumor mutation burden of MPC and non-MPC in TCGA database. (**A,B**) Oncoplot of bladder cancer and skin melanoma (TCGA-BLCA/SKCM). (**C,D**) Tumor mutation burden between MPC and non-MPC in pan-cancer, or in different types of cancer. (**E**) Univariate and multivariate linear regression analysis of history of other primary malignancy and tumor mutation burden.

Although major mutant genes did not significantly differ between MPC and non-MPC in various tumors locations, a higher prevalence of gene mutations was evident in MPC. Notably, bladder urothelial carcinoma (BLCA) and skin cutaneous melanoma (SKCM) were more prone to occur as multiple primary cancers (Fig. 5A,B).

Analysis of the pancancer datasets indicated that the median tumor mutation burden (TMB) with 1.84 mutations/MB (IQR 0.85–4.27) in MPC was significantly higher than that in non-MPC with 1.35 mutations/MB (IQR 0.63–3.20) (p < 0.0001) (Fig. 5C). The differences in TMB here, while statistically significant, are not clinically meaningful. Immunotherapy typically shows limited activity when TMB is less than 2 mutations/MB. In our cohort, there was no significant difference in TMB between MPC (median TMB = 4.30, 32 cases) and non-MPC (median TMB = 4.70, 43 cases) (p > 0.05) (Fig. 4E), maybe primarily attributed to the limited number of cases.

Among 32 different cancers, except for diffuse large B cell lymphoma (DLBC) due to lack of data, 65% (20/31) of the remaining cancer types had a higher mutation load in MPC with a statistically significant difference in five cancers, namely, adrenocortical carcinoma (ACC), cholangiocarcinoma (CHOL), uterine sarcoma (UCS), bladder urothelial carcinoma (BLCA), and skin cutaneous melanoma (SKCM) (all p < 0.05) (Fig. 5D). These findings suggest that a higher TMB when a second or later primary tumor occurs is likely to be a common phenomenon in MPC.

Multivariate analysis revealed a significant correlation between a history of other primary malignancies and a high mutation load with log (TMB + 1) as the dependent variable (B = 0.06, 95% CI 0.01–0.12, p = 0.022) (Fig. 5E). This independent effect may partly be attributed to genetically related tumor syndrome or the influence of radiotherapy and chemotherapy on the treatment of the first primary tumor.

#### Mutant gene features in multiple primary tumors

**Figure 6:**
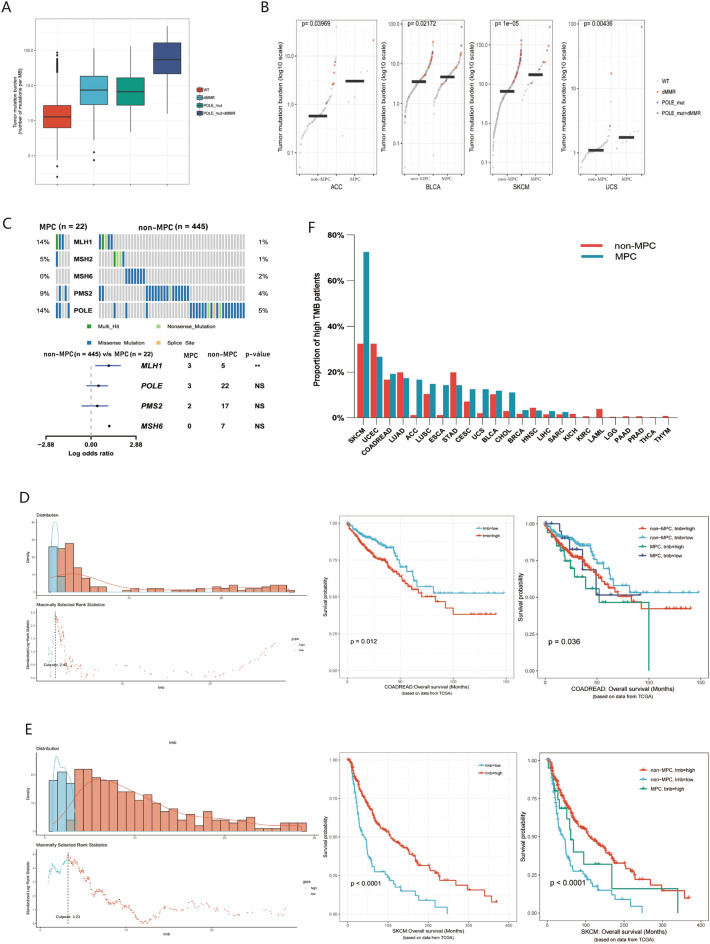
Mutant gene features and prognosis related to TMB in multiple primary tumors. (**A**) Tumor mutation burden and mutation of MMR/POLE gene. (**B**) Tumor mutation burden between MPC and non-MPC in ACC, BLCA, SKCM and UCS. (**C**) Multiple primary cancers have a higher mutation rate of MMR/POLE gene in TCGA-SKCM. (**D,E**) Calculation of cut-off value and Kaplan–Meier survival curve of high TMB and low TMB patients in TCGA-COADREAD/SKCM. (**F**) Proportion of high TMB (> 2.5 mutations/MB) patients in MPC and non-MPC in different types of cancer.

Previous studies reported that mutation of mismatch repair (MMR) genes and the polymerase-epsilon (POLE) gene leads to a significant increase in TMB. The tumor with mismatch repair deficiency (dMMR) and POLE gene mutations exhibit higher TMB, with a further increase when both the MMR and POLE genes are mutated (Fig. 6A). This trend holds across various mutation loads in specific tumors adrenocortical carcinoma (ACC), uterine sarcoma (UCS), bladder urothelial carcinoma (BLCA), and skin cutaneous melanoma (SKCM), irrespective of MPC or non-MPC status (Fig. 6B).

Next, we investigated the mutation ratio of common somatic gene variations in clinical patients between MPC and non-MPC (Fig. 4F). In our cohort, the percentage of gene variations of PIK3CA, BRCA, MSH6 and programmed cell death ligand 1 (PD-L1) (CPS > 1) in MPC was significantly higher than that of non-MPC (p < 0.01 or p < 0.0001) (Fig. 4F). Also, we observed a noticeable increase in microsatellite instability-high (MSI) or MSI-high (MSI-H) patients with MPC than non-MPC (8.8% vs 5.4%, 5.9% vs 3.2%) (Fig. 4F), although not statistically significant. Furthermore, the mutation rate of the MLH1 gene in MPC was significantly higher than that in non-MPC (14% vs. 1%, p < 0.05) in TCGA-SKCM, but there was no significant difference in the POLE and PMS2 genes (Fig. 6C). Similarly significant results were not identified in other tumors.

#### Tumor mutation burden and prognosis in multiple primary tumors

To assess the clinical value of MPC and TMB, both were analyzed as prognostic indicators affecting OS. Combined with the standards in the published literature^12^, > 100 mutations/exome (equivalent to > 2.5 mutations/MB in this study) in TCGA database is the cutoff value of high mutation load for pancancer analysis. The cutoff value for high mutation load was determined based on TMB distribution across different cancers. The impact of mutation load on prognosis varied significantly among different tumors. For the colorectal cancer cohort, the optimal cutoff value was 2.42 mutations/MB, and higher TMB was associated with worse overall survival (Fig. 6D). Nevertheless, in skin cutaneous melanoma (SKCM) with an optimal cutoff of 3.23 mutations/MB, patients with higher TMB had better survival (Fig. 6E). Given the positive response to immune checkpoint inhibitors (ICIs) treatment in individuals with high TMB in recent studies, further research is needed to explore the optimal cutoff value of TMB in various cancers for immunotherapy.

Additionally, we integrated the difference in the proportion of patients with high TMB between the MPC and non-MPC groups. In SKCM, a high mutation load was as high as 72.7%, compared to only 32.4% in the non-MPC group, showing a significant difference between the two groups (p < 0.001) (Fig. 6F). Similarly, 14.3% of patients with secondary primary esophageal cancer (ESCA) had a high mutation load, while 1.2% were in non-MPC patients (p = 0.03) (Fig. 6F). No significant difference was observed in other cancers. Thus, the history of other tumors conveniently obtained by medical history inquiry, could serve a predictor of potential effectiveness for immunotherapy, particularly in SKCM patients.

## Discussion

The survival time of cancer patients worldwide is increasing, and the number of patients with MPC has increased in recent decades^3,5,13^, with a high incidence of 8.5–13.1% since 2020 reported in the study. Compared to the general population, cancer survivors are at much higher risk of SPC and have a poor prognosis^3,5^. The potential risk factors for MPC may include genetic factors, exposure to lifestyle, hormonal factors, immunodeficiency, infection, carcinogenesis of previous iatrogenic treatment, and even the synergistic effect among the above factors^13–17^. And some cancers in same or different sites always share common risk factors but experience different degrees of exposure. In recent decades, research has identified the genetic features of many types of tumors, indicating that approximately 100 genes are prone to one or more cancers when various mutations occur^14^. Consequently, a clear correlation always exists between the occurrence of multiple primary cancers and high genetic mutation load in certain cancers.

Elderly age, male, early tumors, and African black race and white race were independent risk factors for MPC reported in the study. Elderly age itself is the most important risk factor for any kind of cancer, which may be related to the cumulative effect of a variety of risk factors in the long term. The present study also found that some young cancer patients had a high incidence of MPC (such as PCPG and OV), which may be closely related to genetic susceptibility or cancer syndromes^11,16^. The some patients with MPC (especially skin cutaneous melanoma and KIRC) was always found in stage I, suggesting that follow-up was crucial for early detection of the second primary tumor. Except for gender-specific cancers, male cancer patients have a significantly higher incidence of MPC, especially BLCA, mainly due to that smokers are four times more likely to develop bladder cancer than people who never smoke^18^. A previous study has reported that ethnic differences in MPC may be related to genetic and environmental differences^3^.

The age at diagnosis of first primary cancer (FPC) in SMPC patients is notably younger, averaging approximately 10 years less than that in MMPC patients^19,20^. In MMPC, the incidence peak of SPC gradually decreases within 1–5 years after the diagnosis of FPC, with a second smaller peak emerging around 10–15 years. Studies have identified a strong correlation between specific types of first and second primary cancer^5^. For instance, Swiss men and women with oropharyngeal cancer face a 20-fold and 40-fold increased risk of subsequent diagnoses of pharynx cancer and a 16-fold and 30-fold increased risk of developing second primary esophageal cancer, respectively^5^. The combination of FPC and SPC in lung and breast cancer is statistically more significant than in other cancer types (p < 0.0001)^3^. Additionally, bladder cancer is one of the most common SPC, especially when the FPC is renal pelvis and ureter cancer^2^. In summary, gaining a deeper understanding of the clinical characteristics of multiple primary tumors emphasizes the importance of reasonable follow-up for the benefit of cancer patients.

At present, cancer immunotherapy is undergoing rapid development, and TMB has emerged as an important indicator of immunotherapy responsiveness in certain cancers^21^. Pancancer data analysis revealed a significantly higher median TMB of 1.84 mutations/MB in MPC compared to non-MPC at 1.35 mutations/MB (p < 0.001). While statistically significant, these differences may lack clinical significance. In most clinical trials, patients with high TMB (> 10 mutations/MB) significantly benefit from immunotherapy^22^. However, specific cancer types within the MPC group, exhibit significantly higher TMB (> 10 mutations/MB), especially SKCM that reaching a median of 17.31 mutations/MB, in contrast to the non-MPC group at 6.55 mutations/MB. Moreover, targeted agents and immunotherapy significantly optimize outcomes in melanoma, and the median OS of patients with advanced melanoma increased from approximately 9 months before 2011 to at least 2 years^23^. And a high TMB is associated with a clear survival benefit in SKCM. Therefore, a high TMB in MPC may indicate a potential population for immunotherapy.

The present study is subject to several limitations stemming from the retrospective analysis of MPC in registered TCGA database patients. The unavailability of cancer types and diagnostic times for a history of other malignancies prevented the examination of clinical and genomic characteristics of SMPC and MMPC. Despite supplementing data from our medical center, the small sample size hinders the ability to draw normative conclusions, especially without concurrent collection of epidemiological data from the general population. Despite these limitations, the study’s results can serve as a supplementary reference for relevant research.

## Conclusions

In conclusion, MPC is not uncommon and always leads to patients’ poor survival. Some characteristics of MPC requires special attention, encompassing age, gender, staging, race, peaks in the incidence and correlations of location. And MPC always has a higher TMB than non-MPC and associated with a clear survival benefit in certain cancers. A more profound understanding of MPC characteristics is essential for formulating appropriate diagnostic and therapeutic strategies, as well as implementing follow-up protocols in clinical practice.

## Data Availability

The datasets of the patient collected in our center during the current study are available from the corresponding author on reasonable request.
